# Retraction: HIF-1α and HIF-2α: Siblings in promoting angiogenesis of residual hepatocellular carcinoma after high-intensity focused ultrasound ablation

**DOI:** 10.1371/journal.pone.0322422

**Published:** 2025-04-08

**Authors:** The PLOS One Editors

After this article [1] was published, concerns were raised about Figs 2-6 and 8-9.

Specifically:

- The following panels appear to partially overlap:
  ◦ In Fig 2, the 3w panel and the 4w panel.
  ◦ In Fig 3, the 2w panel and the 3w panel.
  ◦ In Fig 4, the control panel and the 1w panel.
  ◦ In Fig 5, the 3d panel and the 3w panel.
  ◦ In Fig 6, the 1w panel and the 2w panel.
- The β-actin panel of Fig 9 appears similar to the β-actin panel of Fig 8 when flipped horizontally and vertically.
- The following panels in [1] and [2] appear to partially overlap:
  ◦ The control panel of Fig 6 in [1] and the control mimic – MVD panel in Figure 3 of [2].
  ◦ The 1d panel of Fig 6 in [1] and the sunitinib+vehicle - MVD panel in Figure 3 of [2].
  ◦ The 3d panel of Fig 6 in [1] and the sunitinib+ADM22-52 - MVD panel in Figure 3 of [2].

In editorial follow-up, the corresponding author (SL) provided updated versions of Figs 2-6, but stated that the original images and quantitative data underlying all figures in [1] are unavailable. The replacement panels included in the updated figures raised further concerns about the reliability of these data. As such, the above concerns remain unresolved.

In light of the above unresolved concerns, which undermine the reliability of the reported results and conclusions, the *PLOS One* Editors retract this article.

SL did not respond to the final editorial decision. LW, ZF, SZ, JG, CAL, and ZQ either did not respond directly or could not be reached.
